# 2-(4-Meth­oxy­phen­yl)-4,5-diphenyl-1-(prop-2-en-1-yl)-1*H*-imidazole

**DOI:** 10.1107/S1600536812041979

**Published:** 2012-10-13

**Authors:** Mehmet Akkurt, Adel A. Marzouk, Vagif. M. Abbasov, Antar A. Abdelhamid, Atash V. Gurbanov

**Affiliations:** a Department of Physics, Faculty of Sciences, Erciyes University, 38039 Kayseri, Turkey; b Pharmaceutical Chemistry Department, Faculty of Pharmacy, Al Azhar University, Egypt; c Mamedaliev Institute of Petrochemical Processes, National Academy of Sciences of Azerbaijan, Baku, Azerbaijan; d Department of Organic Chemistry, Baku State University, Baku, Azerbaijan

## Abstract

The asymmetric unit of the title compound, C_25_H_22_N_2_O, contains two independent mol­ecules (*A* and *B*), with significantly different conformations. In mol­ecule *A*, the central imidazole ring makes dihedral angles of 88.26 (10) and 12.74 (11)° with the two phenyl rings, and 22.06 (9)° with the benzene ring. In mol­ecule *B*, one of the phenyl rings is disordered over two sites, each having an occupancy of 0.5. Here the central imidazole ring forms dihedral angles of 79.24 (10)° with the ordered phenyl ring, and 3.5 (5) and 22.6 (5)° with the two parts of the disordered phenyl ring. The dihedral angle involving the benzene ring is 67.49 (10)°. The —N—C(H_2_)—C(H)—C(H_2_) torsion angles of the prop-1-ene group in the two mol­ecules are very similar, 0.5 (3) and 1.3 (4)° for mol­ecules *A* and *B*, respectively. The crystal structure is stabilized by C—H⋯π inter­actions.

## Related literature

For the synthesis, see: Mohamed *et al.* (2012 ▶). For biological properties of imidazoles, see: Puratchikody & Doble (2007 ▶); Bhatnagar *et al.* (2011 ▶); Antolini *et al.* (1999 ▶); Wang *et al.* (2002 ▶). For standard bond-length data, see: Allen *et al.* (1987 ▶).
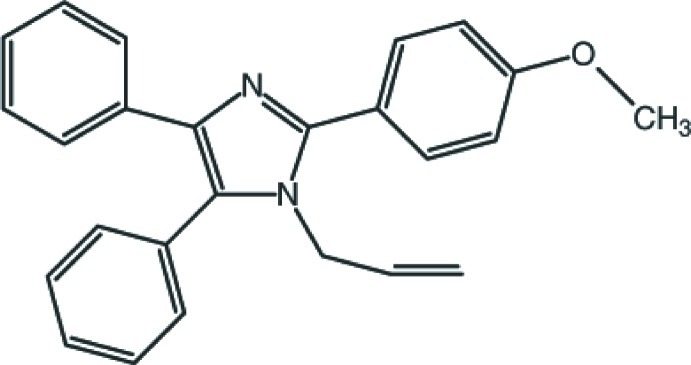


## Experimental

### 

#### Crystal data

C_25_H_22_N_2_O
*M* *_r_* = 366.45
Monoclinic, 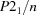

*a* = 18.3169 (7) Å
*b* = 9.6142 (3) Å
*c* = 23.1656 (8) Å
β = 99.0261 (7)°
*V* = 4029.0 (2) Å^3^
*Z* = 8
Mo *K*α radiation
μ = 0.07 mm^−1^
*T* = 296 K
0.30 × 0.30 × 0.20 mm

#### Data collection

Bruker APEXII CCD diffractometer
Absorption correction: multi-scan (*SADABS*; Sheldrick, 2003 ▶) *T*_min_ = 0.978, *T*_max_ = 0.985
44087 measured reflections
9608 independent reflections
6879 reflections with *I* > 2σ(*I*)
*R*_int_ = 0.026

#### Refinement

*R*[*F* ^2^ > 2σ(*F* ^2^)] = 0.058
*wR*(*F* ^2^) = 0.181
*S* = 1.01
9608 reflections
511 parameters
37 restraints
H-atom parameters constrained
Δρ_max_ = 0.60 e Å^−3^
Δρ_min_ = −0.39 e Å^−3^

### 

Data collection: *APEX2* (Bruker, 2005 ▶); cell refinement: *SAINT-Plus* (Bruker, 2001 ▶); data reduction: *SAINT-Plus*; program(s) used to solve structure: *SHELXS97* (Sheldrick, 2008 ▶); program(s) used to refine structure: *SHELXL97* (Sheldrick, 2008 ▶); molecular graphics: *ORTEP-3 for Windows* (Farrugia, 2012 ▶); software used to prepare material for publication: *WinGX* (Farrugia, 2012 ▶) and *PLATON* (Spek, 2009 ▶).

## Supplementary Material

Crystal structure: contains datablock(s) global, I. DOI: 10.1107/S1600536812041979/su2509sup1.cif

Structure factors: contains datablock(s) I. DOI: 10.1107/S1600536812041979/su2509Isup2.hkl

Supplementary material file. DOI: 10.1107/S1600536812041979/su2509Isup3.cml

Additional supplementary materials:  crystallographic information; 3D view; checkCIF report

## Figures and Tables

**Table 1:** Hydrogen-bond geometry (Å, °) *Cg*1, *Cg*2, *Cg*3, *Cg*4 and *Cg*5 are the centroids of the N3/N4/C29–C31, C38′–C43′, C7–C12, C13–C18 and C19-C24 rings, respectively.

*D*—H⋯*A*	*D*—H	H⋯*A*	*D*⋯*A*	*D*—H⋯*A*
C8—H8⋯*Cg*5^i^	0.93	2.87	3.585 (2)	135
C28—H28*A*⋯*Cg*2^ii^	0.97	2.99	3.655 (6)	127
C33—H33⋯*Cg*1^iii^	0.93	2.86	3.592 (2)	137
C37—H37⋯*Cg*1^ii^	0.93	2.96	3.789 (2)	150
C41—H41⋯*Cg*3^iv^	0.93	2.90	3.772 (10)	157
C41′—H41′⋯*Cg*3^iv^	0.93	2.85	3.705 (12)	154
C46—H46⋯*Cg*4	0.93	3.00	3.712 (2)	135

Symmetry codes: (i) 
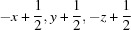
; (ii) 
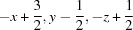
; (iii) 
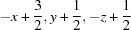
; (iv) 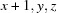.

**Fig. 1.**
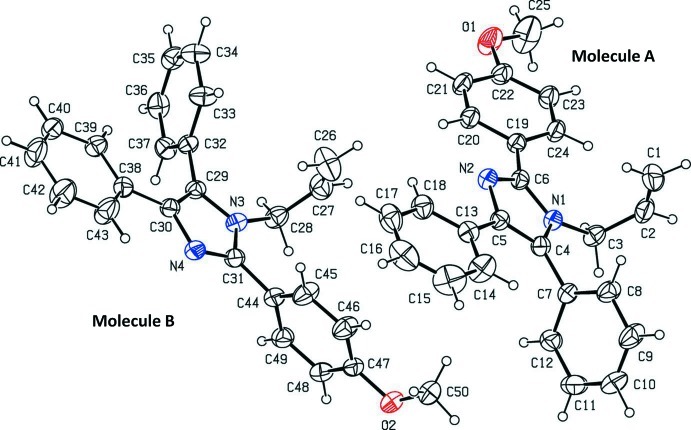
A view of the two independent molecules (A and B) of the title compound, with the atom numbering. Displacement ellipsoids are drawn at the 30% probability level. In molecule B only one of the disordered parts is shown.

**Fig. 2.**
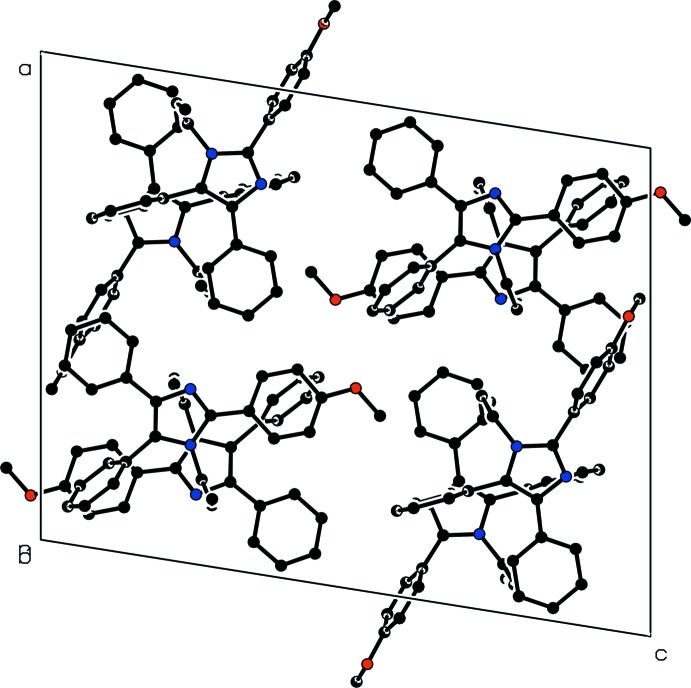
A view along the b axis of the crystal packing of the title compound. H atoms have been omitted for clarity.

## supplementary crystallographic information

### Comment

Imidazole derivatives are a great class of heterocyclic compounds due to their wide range of pharmacological and biological activities They exhibited as inhibitors of p38 MAP kinase, fungicides, herbicides, plant growth regulators, antibacterial, antitumour, pesticides and therapeutic agents (Bhatnagar *et al*. (2011); Puratchikody & Doble 2007; Wang *et al.*, 2002 and Antolini, *et al.*, 1999). They also serve as useful building blocks for synthesis of diverse class of bioactive molecules. Further to our study on synthesis of bioactive heterocyclic molecules we herein report the crystal structure of the title compound.

The molecular structure of the two independent molecules (A and B) of the title compound are illustrated in Fig. 1 Both molecules have a similar conformation. The –N—C(H_2_)—C(H)—C(H_2_) torsion angles of the prop-1-ene group are 0.5 (3) and 1.3 (4)° in molecules A and B, respectively. The bond lengths and angles of both molecules are comparable and similar to the values reported in the literature (Allen *et al.*, 1987).

In molecule A, the central imidazole ring (N1/N2/C4–C6) forms dihedral angles of 88.26 (10), 12.74 (11) and 22.06 (9)° with the C7–C12 and C13–C18 phenyl and C19—C24 benzene rings, respectively. The corresponding angles in molecule B are 79.24 (10), 3.5 (5) [22.6 (5)] and 67.49 (10)°, with the C32–C37 and disordered C38–C43 [C38'–C43'] phenyl and C44—C49 benzene rings, respectively. In the disordered phenyl group of molecule B, the dihedral angle between the two parts is 19.1 (7)°.

In the crystal, there is no classical hydrogen bonds; the molecules are linked via C—H···π interactions (Table 1 and Fig. 2).

### Experimental

The title compound was prepared, among series of imidazole derivatives, according to our reported method (Mohamed *et al.*, 2012) in 88% yield. Suitable single crystals were obtained by slow evaporation of a solution in ethanol *M*.p. 385–387 K.

### Refinement

H atoms were positioned geometrically, C—H = 0.93 Å (aromatic), 0.96 Å (methyl) and 0.97 Å (methylene) H atoms, respectively, and refined as riding with *U*_iso_(H) = 1.2*U*_eq_(C) or 1.5*U*_eq_(C) for methyl H atoms. Each atom of the disordered phenyl ring [one part C38–C43 and the other C38'–C43'] of molecule B have an occupancy of 0.5. Their displacement parameters were restrained using ISOR. The C30—C38 and C30—C38' distances were restrained to be equal using the command SADI. Their anisotropic displacement parameters were also made equal using the EADP constraint. Two reflections, (0 2 3) and (-1 0 1), were omitted owing interference from the beam stop.

### Crystal data

**Table:**

C_25_H_22_N_2_O	*F*(000) = 1552
*M**_r_* = 366.45	*D*_x_ = 1.208 Mg m^−^^3^
Monoclinic, *P*2_1_/*n*	Mo *K*α radiation, λ = 0.71073 Å
Hall symbol: -P 2yn	Cell parameters from 9921 reflections
*a* = 18.3169 (7) Å	θ = 2.3–28.8°
*b* = 9.6142 (3) Å	µ = 0.07 mm^−^^1^
*c* = 23.1656 (8) Å	*T* = 296 K
β = 99.0261 (7)°	Prism, colourless
*V* = 4029.0 (2) Å^3^	0.30 × 0.30 × 0.20 mm
*Z* = 8	

### Data collection

**Table:**

Bruker APEXII CCD diffractometer	9608 independent reflections
Radiation source: fine-focus sealed tube	6879 reflections with *I* > 2σ(*I*)
Graphite monochromator	*R*_int_ = 0.026
φ and ω scans	θ_max_ = 27.5°, θ_min_ = 1.5°
Absorption correction: multi-scan (*SADABS*; Sheldrick, 2003)	*h* = −24→24
*T*_min_ = 0.978, *T*_max_ = 0.985	*k* = −12→12
44087 measured reflections	*l* = −30→30

### Refinement

**Table:**

Refinement on *F*^2^	Primary atom site location: structure-invariant direct methods
Least-squares matrix: full	Secondary atom site location: difference Fourier map
*R*[*F*^2^ > 2σ(*F*^2^)] = 0.058	Hydrogen site location: difference Fourier map
*wR*(*F*^2^) = 0.181	H-atom parameters constrained
*S* = 1.01	*w* = 1/[σ^2^(*F*_o_^2^) + (0.088*P*)^2^ + 1.3785*P*] where *P* = (*F*_o_^2^ + 2*F*_c_^2^)/3
9608 reflections	(Δ/σ)_max_ < 0.001
511 parameters	Δρ_max_ = 0.60 e Å^−^^3^
37 restraints	Δρ_min_ = −0.39 e Å^−^^3^

### Special details

**Table:**

Geometry. Bond distances, angles *etc*. have been calculated using the rounded fractional coordinates. All su's are estimated from the variances of the (full) variance-covariance matrix. The cell e.s.d.'s are taken into account in the estimation of distances, angles and torsion angles
Refinement. Refinement on *F*^2^ for ALL reflections except those flagged by the user for potential systematic errors. Weighted *R*-factors *wR* and all goodnesses of fit *S* are based on *F*^2^, conventional *R*-factors *R* are based on *F*, with *F* set to zero for negative *F*^2^. The observed criterion of *F*^2^ > σ(*F*^2^) is used only for calculating -*R*-factor-obs *etc*. and is not relevant to the choice of reflections for refinement. *R*-factors based on *F*^2^ are statistically about twice as large as those based on *F*, and *R*-factors based on ALL data will be even larger.

### Fractional atomic coordinates and isotropic or equivalent isotropic displacement parameters (Å^2^)

**Table:**

	*x*	*y*	*z*	*U*_iso_*/*U*_eq_	Occ. (<1)
O2	0.35130 (8)	0.31803 (17)	0.03555 (7)	0.0694 (5)	
N3	0.65418 (8)	0.34591 (15)	0.21941 (6)	0.0476 (5)	
N4	0.69985 (8)	0.39192 (16)	0.13852 (6)	0.0494 (5)	
C26	0.57202 (19)	0.5661 (3)	0.26307 (15)	0.1047 (14)	
C27	0.56204 (13)	0.4411 (3)	0.27676 (11)	0.0761 (9)	
C28	0.60026 (11)	0.3178 (2)	0.25849 (9)	0.0593 (7)	
C29	0.72817 (10)	0.37696 (17)	0.23660 (8)	0.0447 (5)	
C30	0.75584 (10)	0.40284 (17)	0.18608 (7)	0.0450 (5)	
C31	0.63998 (10)	0.35807 (18)	0.16008 (8)	0.0471 (5)	
C32	0.76131 (9)	0.38301 (17)	0.29897 (7)	0.0435 (5)	
C33	0.77225 (13)	0.5095 (2)	0.32740 (8)	0.0603 (7)	
C34	0.80412 (13)	0.5164 (2)	0.38539 (9)	0.0672 (7)	
C35	0.82503 (11)	0.3971 (3)	0.41585 (9)	0.0623 (7)	
C36	0.81407 (12)	0.2707 (2)	0.38842 (9)	0.0615 (7)	
C37	0.78263 (11)	0.2638 (2)	0.33034 (9)	0.0533 (6)	
C38	0.8320 (4)	0.4410 (18)	0.1789 (7)	0.0509 (7)	0.500
C38'	0.8312 (5)	0.4382 (18)	0.1761 (7)	0.0509 (7)	0.500
C39	0.8898 (7)	0.4463 (14)	0.2255 (5)	0.0545 (17)	0.500
C39'	0.8940 (7)	0.4217 (15)	0.2157 (5)	0.0545 (17)	0.500
C40	0.9605 (5)	0.4819 (14)	0.2159 (5)	0.0590 (19)	0.500
C40'	0.9631 (6)	0.4565 (14)	0.2053 (5)	0.0590 (19)	0.500
C41	0.9733 (4)	0.5123 (16)	0.1597 (6)	0.0922 (13)	0.500
C41'	0.9742 (5)	0.5161 (18)	0.1565 (7)	0.0922 (13)	0.500
C42	0.9155 (7)	0.5070 (13)	0.1131 (5)	0.097 (3)	0.500
C42'	0.9129 (7)	0.5598 (14)	0.1165 (5)	0.097 (3)	0.500
C43	0.8448 (5)	0.4713 (15)	0.1227 (6)	0.089 (3)	0.500
C43'	0.8435 (6)	0.5163 (16)	0.1255 (6)	0.089 (3)	0.500
C44	0.56529 (10)	0.34450 (19)	0.12565 (8)	0.0492 (5)	
C45	0.53132 (12)	0.4605 (2)	0.09929 (10)	0.0683 (7)	
C46	0.46031 (12)	0.4561 (2)	0.06839 (10)	0.0687 (7)	
C47	0.42230 (10)	0.3326 (2)	0.06372 (8)	0.0525 (6)	
C48	0.45600 (11)	0.2144 (2)	0.08847 (9)	0.0590 (7)	
C49	0.52640 (11)	0.2200 (2)	0.11945 (9)	0.0567 (6)	
C50	0.31184 (13)	0.4411 (3)	0.01802 (12)	0.0837 (9)	
O1	0.41271 (8)	0.5961 (2)	0.51655 (6)	0.0853 (7)	
N1	0.24289 (8)	0.67256 (15)	0.24563 (6)	0.0439 (4)	
N2	0.35708 (8)	0.74733 (15)	0.24549 (6)	0.0467 (4)	
C1	0.13887 (15)	0.8646 (3)	0.28108 (12)	0.0839 (10)	
C2	0.12641 (11)	0.7324 (3)	0.28092 (10)	0.0685 (8)	
C3	0.17539 (10)	0.6195 (2)	0.26386 (9)	0.0537 (6)	
C4	0.24870 (9)	0.70292 (17)	0.18810 (8)	0.0449 (5)	
C5	0.32016 (9)	0.74795 (18)	0.18908 (8)	0.0447 (5)	
C6	0.30977 (9)	0.70180 (17)	0.27875 (7)	0.0434 (5)	
C7	0.18709 (9)	0.67909 (18)	0.13956 (8)	0.0460 (5)	
C8	0.13833 (11)	0.7848 (2)	0.11944 (10)	0.0652 (7)	
C9	0.08342 (13)	0.7631 (3)	0.07206 (12)	0.0778 (9)	
C10	0.07678 (13)	0.6373 (3)	0.04448 (10)	0.0733 (8)	
C11	0.12369 (14)	0.5311 (3)	0.06443 (10)	0.0734 (8)	
C12	0.17867 (12)	0.5519 (2)	0.11174 (9)	0.0597 (6)	
C13	0.35943 (10)	0.78571 (19)	0.14044 (8)	0.0496 (6)	
C14	0.32434 (15)	0.8064 (3)	0.08369 (10)	0.0784 (9)	
C15	0.3645 (2)	0.8332 (3)	0.03948 (12)	0.0982 (13)	
C16	0.43927 (19)	0.8436 (3)	0.05000 (13)	0.0902 (13)	
C17	0.47490 (15)	0.8273 (3)	0.10583 (13)	0.0791 (10)	
C18	0.43551 (12)	0.7975 (2)	0.15076 (10)	0.0626 (7)	
C19	0.33115 (9)	0.67911 (18)	0.34176 (8)	0.0448 (5)	
C20	0.40664 (10)	0.6643 (2)	0.36323 (8)	0.0537 (6)	
C21	0.43180 (10)	0.6392 (2)	0.42109 (9)	0.0593 (6)	
C22	0.38231 (11)	0.6259 (2)	0.46035 (8)	0.0576 (6)	
C23	0.30776 (10)	0.6440 (2)	0.44088 (8)	0.0579 (7)	
C24	0.28301 (10)	0.6711 (2)	0.38261 (8)	0.0525 (6)	
C25	0.36398 (18)	0.5660 (5)	0.55645 (12)	0.1319 (18)	
H26A	0.60660	0.58750	0.23900	0.1260*	
H26B	0.54480	0.63670	0.27710	0.1260*	
H27	0.52690	0.42530	0.30090	0.0910*	
H28A	0.62550	0.27160	0.29320	0.0710*	
H28B	0.56340	0.25390	0.23900	0.0710*	
H33	0.75790	0.59110	0.30720	0.0720*	
H34	0.81140	0.60230	0.40380	0.0810*	
H35	0.84660	0.40180	0.45490	0.0750*	
H36	0.82790	0.18950	0.40900	0.0740*	
H37	0.77570	0.17760	0.31210	0.0640*	
H39	0.88130	0.42600	0.26310	0.0650*	0.500
H39'	0.88950	0.38450	0.25200	0.0650*	0.500
H40	0.99920	0.48550	0.24710	0.0710*	0.500
H40'	1.00360	0.43720	0.23380	0.0710*	0.500
H41	1.02060	0.53610	0.15320	0.1100*	0.500
H41'	1.02190	0.52920	0.14860	0.1100*	0.500
H42	0.92400	0.52730	0.07540	0.1160*	0.500
H42'	0.91870	0.61610	0.08490	0.1160*	0.500
H43	0.80610	0.46780	0.09150	0.1070*	0.500
H43'	0.80290	0.53860	0.09760	0.1070*	0.500
H45	0.55680	0.54450	0.10230	0.0820*	
H46	0.43850	0.53620	0.05090	0.0820*	
H48	0.43100	0.12990	0.08420	0.0710*	
H49	0.54820	0.13950	0.13650	0.0680*	
H50A	0.26310	0.41760	−0.00130	0.1250*	
H50B	0.33730	0.49220	−0.00830	0.1250*	
H50C	0.30820	0.49710	0.05180	0.1250*	
H1A	0.18210	0.89830	0.26980	0.1010*	
H1B	0.10470	0.92610	0.29250	0.1010*	
H2	0.08240	0.70420	0.29260	0.0820*	
H3A	0.18860	0.55720	0.29680	0.0640*	
H3B	0.14810	0.56600	0.23210	0.0640*	
H8	0.14250	0.87100	0.13790	0.0780*	
H9	0.05080	0.83460	0.05890	0.0930*	
H10	0.04040	0.62380	0.01210	0.0880*	
H11	0.11860	0.44480	0.04610	0.0880*	
H12	0.21040	0.47920	0.12500	0.0720*	
H14	0.27300	0.80210	0.07530	0.0940*	
H15	0.33990	0.84440	0.00150	0.1180*	
H16	0.46570	0.86150	0.01970	0.1080*	
H17	0.52600	0.83620	0.11380	0.0950*	
H18	0.46070	0.78520	0.18850	0.0750*	
H20	0.44060	0.67170	0.33740	0.0640*	
H21	0.48230	0.63100	0.43410	0.0710*	
H23	0.27420	0.63790	0.46700	0.0690*	
H24	0.23270	0.68450	0.37020	0.0630*	
H25A	0.39200	0.54800	0.59430	0.1980*	
H25B	0.33500	0.48570	0.54330	0.1980*	
H25C	0.33190	0.64410	0.55880	0.1980*	

### Atomic displacement parameters (Å^2^)

**Table:**

	*U*^11^	*U*^22^	*U*^33^	*U*^12^	*U*^13^	*U*^23^
O2	0.0490 (8)	0.0850 (11)	0.0688 (9)	−0.0031 (7)	−0.0075 (7)	−0.0143 (8)
N3	0.0471 (8)	0.0474 (8)	0.0463 (8)	−0.0054 (6)	0.0015 (6)	−0.0007 (6)
N4	0.0473 (8)	0.0524 (8)	0.0464 (8)	0.0008 (6)	0.0007 (6)	−0.0031 (6)
C26	0.114 (2)	0.085 (2)	0.127 (3)	0.0191 (17)	0.056 (2)	0.0053 (18)
C27	0.0659 (14)	0.0991 (19)	0.0663 (14)	0.0022 (13)	0.0199 (11)	−0.0006 (13)
C28	0.0536 (11)	0.0711 (13)	0.0527 (11)	−0.0136 (9)	0.0070 (8)	0.0046 (9)
C29	0.0467 (9)	0.0371 (8)	0.0481 (9)	−0.0014 (7)	0.0010 (7)	−0.0012 (7)
C30	0.0464 (9)	0.0415 (8)	0.0452 (9)	0.0026 (7)	0.0009 (7)	−0.0020 (7)
C31	0.0476 (9)	0.0447 (9)	0.0471 (9)	−0.0002 (7)	0.0018 (7)	−0.0038 (7)
C32	0.0437 (8)	0.0422 (8)	0.0433 (8)	−0.0041 (7)	0.0029 (7)	0.0030 (7)
C33	0.0883 (15)	0.0410 (9)	0.0477 (10)	−0.0088 (9)	−0.0010 (10)	0.0042 (8)
C34	0.0916 (16)	0.0606 (12)	0.0475 (10)	−0.0196 (11)	0.0047 (10)	−0.0042 (9)
C35	0.0567 (11)	0.0866 (15)	0.0419 (9)	−0.0046 (10)	0.0024 (8)	0.0066 (10)
C36	0.0623 (12)	0.0677 (13)	0.0545 (11)	0.0161 (10)	0.0088 (9)	0.0204 (10)
C37	0.0601 (11)	0.0434 (9)	0.0565 (11)	0.0055 (8)	0.0096 (9)	0.0045 (8)
C38	0.0468 (9)	0.0560 (11)	0.0495 (14)	0.0054 (8)	0.0060 (8)	−0.0034 (10)
C38'	0.0468 (9)	0.0560 (11)	0.0495 (14)	0.0054 (8)	0.0060 (8)	−0.0034 (10)
C39	0.0537 (16)	0.059 (4)	0.050 (3)	−0.005 (2)	0.0055 (17)	0.002 (3)
C39'	0.0537 (16)	0.059 (4)	0.050 (3)	−0.005 (2)	0.0055 (17)	0.002 (3)
C40	0.0494 (12)	0.062 (4)	0.063 (4)	−0.0012 (17)	0.0008 (17)	−0.002 (3)
C40'	0.0494 (12)	0.062 (4)	0.063 (4)	−0.0012 (17)	0.0008 (17)	−0.002 (3)
C41	0.0475 (12)	0.155 (3)	0.076 (2)	−0.0038 (15)	0.0157 (11)	−0.0006 (18)
C41'	0.0475 (12)	0.155 (3)	0.076 (2)	−0.0038 (15)	0.0157 (11)	−0.0006 (18)
C42	0.0684 (17)	0.164 (9)	0.0612 (18)	−0.014 (4)	0.0200 (14)	0.000 (4)
C42'	0.0684 (17)	0.164 (9)	0.0612 (18)	−0.014 (4)	0.0200 (14)	0.000 (4)
C43	0.0554 (13)	0.160 (8)	0.0508 (16)	−0.010 (3)	0.0063 (11)	−0.004 (4)
C43'	0.0554 (13)	0.160 (8)	0.0508 (16)	−0.010 (3)	0.0063 (11)	−0.004 (4)
C44	0.0468 (9)	0.0524 (10)	0.0463 (9)	−0.0024 (8)	0.0007 (7)	−0.0043 (8)
C45	0.0620 (12)	0.0536 (11)	0.0813 (15)	−0.0116 (9)	−0.0135 (11)	0.0102 (10)
C46	0.0626 (12)	0.0589 (12)	0.0763 (14)	−0.0019 (10)	−0.0146 (11)	0.0088 (11)
C47	0.0454 (9)	0.0670 (12)	0.0437 (9)	−0.0036 (8)	0.0027 (7)	−0.0118 (8)
C48	0.0563 (11)	0.0535 (11)	0.0649 (12)	−0.0104 (9)	0.0028 (9)	−0.0119 (9)
C49	0.0562 (11)	0.0491 (10)	0.0623 (12)	−0.0007 (8)	0.0012 (9)	−0.0047 (9)
C50	0.0544 (12)	0.106 (2)	0.0837 (16)	0.0134 (13)	−0.0106 (11)	−0.0220 (15)
O1	0.0573 (9)	0.1517 (17)	0.0435 (8)	0.0046 (10)	−0.0027 (6)	0.0062 (9)
N1	0.0393 (7)	0.0465 (8)	0.0453 (7)	0.0014 (6)	0.0050 (6)	0.0036 (6)
N2	0.0417 (7)	0.0500 (8)	0.0476 (8)	0.0008 (6)	0.0049 (6)	0.0033 (6)
C1	0.0707 (15)	0.0872 (19)	0.0921 (18)	0.0264 (13)	0.0075 (13)	−0.0095 (14)
C2	0.0370 (9)	0.1041 (19)	0.0649 (13)	0.0000 (10)	0.0093 (9)	−0.0013 (12)
C3	0.0464 (9)	0.0589 (11)	0.0550 (10)	−0.0126 (8)	0.0059 (8)	0.0041 (8)
C4	0.0457 (9)	0.0420 (8)	0.0457 (9)	0.0041 (7)	0.0034 (7)	0.0030 (7)
C5	0.0433 (8)	0.0430 (9)	0.0469 (9)	0.0016 (7)	0.0043 (7)	0.0036 (7)
C6	0.0388 (8)	0.0429 (8)	0.0477 (9)	0.0022 (7)	0.0048 (7)	0.0016 (7)
C7	0.0421 (9)	0.0488 (9)	0.0460 (9)	−0.0007 (7)	0.0032 (7)	0.0041 (7)
C8	0.0590 (12)	0.0499 (11)	0.0785 (14)	0.0038 (9)	−0.0151 (10)	−0.0015 (10)
C9	0.0599 (13)	0.0715 (15)	0.0908 (17)	0.0033 (11)	−0.0231 (12)	0.0118 (13)
C10	0.0615 (13)	0.0886 (17)	0.0625 (13)	−0.0146 (12)	−0.0126 (10)	0.0015 (12)
C11	0.0801 (15)	0.0712 (14)	0.0647 (13)	−0.0081 (12)	−0.0017 (11)	−0.0190 (11)
C12	0.0610 (11)	0.0561 (11)	0.0600 (11)	0.0058 (9)	0.0029 (9)	−0.0050 (9)
C13	0.0543 (10)	0.0437 (9)	0.0515 (10)	0.0005 (8)	0.0109 (8)	0.0048 (7)
C14	0.0766 (15)	0.0973 (18)	0.0597 (13)	−0.0072 (13)	0.0057 (11)	0.0277 (12)
C15	0.126 (3)	0.111 (2)	0.0597 (14)	−0.0142 (19)	0.0208 (15)	0.0292 (14)
C16	0.126 (3)	0.0722 (16)	0.0857 (19)	−0.0062 (16)	0.0581 (18)	0.0139 (13)
C17	0.0759 (15)	0.0681 (14)	0.103 (2)	−0.0029 (12)	0.0446 (15)	0.0042 (13)
C18	0.0592 (12)	0.0637 (12)	0.0671 (13)	−0.0022 (10)	0.0172 (10)	0.0035 (10)
C19	0.0405 (8)	0.0456 (9)	0.0471 (9)	0.0012 (7)	0.0028 (7)	−0.0003 (7)
C20	0.0401 (9)	0.0699 (12)	0.0514 (10)	−0.0025 (8)	0.0082 (7)	−0.0012 (9)
C21	0.0375 (9)	0.0860 (14)	0.0516 (10)	−0.0002 (9)	−0.0019 (8)	−0.0033 (10)
C22	0.0491 (10)	0.0773 (13)	0.0437 (9)	0.0007 (9)	−0.0009 (8)	−0.0021 (9)
C23	0.0454 (10)	0.0815 (14)	0.0470 (10)	0.0019 (9)	0.0081 (8)	0.0015 (9)
C24	0.0366 (8)	0.0694 (12)	0.0500 (10)	0.0045 (8)	0.0022 (7)	0.0023 (9)
C25	0.0859 (19)	0.259 (5)	0.0510 (14)	0.016 (3)	0.0110 (13)	0.032 (2)

### Geometric parameters (Å, º)

**Table:**

O2—C47	1.368 (2)	C41—H41	0.9300
O2—C50	1.413 (3)	C41'—H41'	0.9300
O1—C22	1.364 (2)	C42—H42	0.9300
O1—C25	1.412 (3)	C42'—H42'	0.9300
N3—C31	1.363 (2)	C43—H43	0.9300
N3—C29	1.384 (2)	C43'—H43'	0.9300
N3—C28	1.466 (3)	C45—H45	0.9300
N4—C30	1.386 (2)	C46—H46	0.9300
N4—C31	1.315 (2)	C48—H48	0.9300
N1—C4	1.385 (2)	C49—H49	0.9300
N1—C6	1.369 (2)	C50—H50A	0.9600
N1—C3	1.460 (2)	C50—H50B	0.9600
N2—C5	1.374 (2)	C50—H50C	0.9600
N2—C6	1.321 (2)	C1—C2	1.291 (4)
C26—C27	1.264 (4)	C2—C3	1.500 (3)
C27—C28	1.472 (3)	C4—C5	1.376 (2)
C29—C30	1.369 (2)	C4—C7	1.481 (2)
C29—C32	1.478 (2)	C5—C13	1.474 (3)
C30—C38	1.477 (9)	C6—C19	1.467 (2)
C30—C38'	1.475 (10)	C7—C8	1.385 (3)
C31—C44	1.477 (3)	C7—C12	1.380 (3)
C32—C33	1.383 (3)	C8—C9	1.383 (3)
C32—C37	1.380 (3)	C9—C10	1.364 (4)
C33—C34	1.380 (3)	C10—C11	1.367 (4)
C34—C35	1.369 (3)	C11—C12	1.382 (3)
C35—C36	1.371 (3)	C13—C14	1.384 (3)
C36—C37	1.380 (3)	C13—C18	1.381 (3)
C38—C43	1.39 (2)	C14—C15	1.376 (4)
C38—C39	1.389 (18)	C15—C16	1.357 (5)
C38'—C39'	1.363 (18)	C16—C17	1.363 (4)
C38'—C43'	1.44 (2)	C17—C18	1.386 (4)
C39—C40	1.391 (16)	C19—C20	1.401 (3)
C39'—C40'	1.367 (17)	C19—C24	1.393 (3)
C40—C41	1.390 (18)	C20—C21	1.369 (3)
C40'—C41'	1.31 (2)	C21—C22	1.387 (3)
C41—C42	1.389 (17)	C22—C23	1.380 (3)
C41'—C42'	1.403 (18)	C23—C24	1.380 (3)
C42—C43	1.391 (16)	C1—H1A	0.9300
C42'—C43'	1.385 (17)	C1—H1B	0.9300
C44—C45	1.373 (3)	C2—H2	0.9300
C44—C49	1.389 (3)	C3—H3A	0.9700
C45—C46	1.383 (3)	C3—H3B	0.9700
C46—C47	1.372 (3)	C8—H8	0.9300
C47—C48	1.375 (3)	C9—H9	0.9300
C48—C49	1.375 (3)	C10—H10	0.9300
C26—H26B	0.9300	C11—H11	0.9300
C26—H26A	0.9300	C12—H12	0.9300
C27—H27	0.9300	C14—H14	0.9300
C28—H28B	0.9700	C15—H15	0.9300
C28—H28A	0.9700	C16—H16	0.9300
C33—H33	0.9300	C17—H17	0.9300
C34—H34	0.9300	C18—H18	0.9300
C35—H35	0.9300	C20—H20	0.9300
C36—H36	0.9300	C21—H21	0.9300
C37—H37	0.9300	C23—H23	0.9300
C39—H39	0.9300	C24—H24	0.9300
C39'—H39'	0.9300	C25—H25A	0.9600
C40—H40	0.9300	C25—H25B	0.9600
C40'—H40'	0.9300	C25—H25C	0.9600
			
C47—O2—C50	117.18 (17)	C42'—C43'—H43'	119.00
C22—O1—C25	117.56 (18)	C44—C45—H45	119.00
C28—N3—C29	125.90 (15)	C46—C45—H45	119.00
C28—N3—C31	126.73 (15)	C47—C46—H46	120.00
C29—N3—C31	107.16 (15)	C45—C46—H46	120.00
C30—N4—C31	105.89 (14)	C49—C48—H48	120.00
C3—N1—C6	129.33 (15)	C47—C48—H48	120.00
C3—N1—C4	123.27 (15)	C44—C49—H49	120.00
C4—N1—C6	107.40 (14)	C48—C49—H49	120.00
C5—N2—C6	106.62 (14)	H50A—C50—H50C	109.00
C26—C27—C28	126.9 (3)	H50B—C50—H50C	109.00
N3—C28—C27	115.21 (17)	O2—C50—H50A	109.00
N3—C29—C30	105.69 (15)	O2—C50—H50B	110.00
N3—C29—C32	121.55 (15)	O2—C50—H50C	109.00
C30—C29—C32	132.70 (17)	H50A—C50—H50B	109.00
N4—C30—C38	121.6 (6)	C1—C2—C3	126.9 (2)
N4—C30—C38'	119.1 (6)	N1—C3—C2	113.08 (17)
N4—C30—C29	109.79 (16)	N1—C4—C5	105.49 (15)
C38—C30—C38'	2.7 (9)	N1—C4—C7	122.27 (15)
C29—C30—C38	128.6 (6)	C5—C4—C7	132.16 (17)
C29—C30—C38'	131.1 (6)	N2—C5—C4	109.84 (16)
N4—C31—C44	125.00 (16)	N2—C5—C13	120.11 (15)
N3—C31—C44	123.42 (16)	C4—C5—C13	129.95 (17)
N3—C31—N4	111.44 (16)	N1—C6—N2	110.63 (14)
C29—C32—C37	121.39 (15)	N1—C6—C19	127.08 (15)
C33—C32—C37	118.14 (16)	N2—C6—C19	122.17 (15)
C29—C32—C33	120.46 (15)	C4—C7—C8	121.10 (16)
C32—C33—C34	120.91 (17)	C4—C7—C12	120.53 (16)
C33—C34—C35	120.19 (19)	C8—C7—C12	118.32 (18)
C34—C35—C36	119.65 (19)	C7—C8—C9	120.4 (2)
C35—C36—C37	120.19 (19)	C8—C9—C10	120.4 (2)
C32—C37—C36	120.92 (18)	C9—C10—C11	119.9 (2)
C30—C38—C43	117.3 (10)	C10—C11—C12	120.1 (2)
C30—C38—C39	122.7 (12)	C7—C12—C11	120.9 (2)
C39—C38—C43	120.0 (9)	C5—C13—C14	123.49 (19)
C30—C38'—C39'	125.4 (13)	C5—C13—C18	119.21 (17)
C30—C38'—C43'	121.2 (10)	C14—C13—C18	117.3 (2)
C39'—C38'—C43'	112.7 (10)	C13—C14—C15	120.7 (3)
C38—C39—C40	120.0 (11)	C14—C15—C16	121.5 (3)
C38'—C39'—C40'	124.1 (12)	C15—C16—C17	118.8 (3)
C39—C40—C41	120.0 (10)	C16—C17—C18	120.5 (3)
C39'—C40'—C41'	122.2 (11)	C13—C18—C17	121.1 (2)
C40—C41—C42	120.0 (9)	C6—C19—C20	117.57 (15)
C40'—C41'—C42'	118.9 (10)	C6—C19—C24	125.84 (16)
C41—C42—C43	120.0 (11)	C20—C19—C24	116.60 (17)
C41'—C42'—C43'	117.9 (12)	C19—C20—C21	121.83 (17)
C38—C43—C42	120.0 (11)	C20—C21—C22	120.27 (18)
C38'—C43'—C42'	123.0 (11)	O1—C22—C21	115.75 (18)
C31—C44—C45	119.02 (17)	O1—C22—C23	124.98 (17)
C45—C44—C49	117.70 (18)	C21—C22—C23	119.27 (17)
C31—C44—C49	123.26 (17)	C22—C23—C24	119.99 (17)
C44—C45—C46	121.94 (18)	C19—C24—C23	121.95 (17)
C45—C46—C47	119.44 (18)	C2—C1—H1A	120.00
C46—C47—C48	119.56 (18)	C2—C1—H1B	120.00
O2—C47—C46	123.99 (18)	H1A—C1—H1B	120.00
O2—C47—C48	116.45 (17)	C1—C2—H2	117.00
C47—C48—C49	120.55 (18)	C3—C2—H2	117.00
C44—C49—C48	120.77 (18)	N1—C3—H3A	109.00
C27—C26—H26A	120.00	N1—C3—H3B	109.00
H26A—C26—H26B	120.00	C2—C3—H3A	109.00
C27—C26—H26B	120.00	C2—C3—H3B	109.00
C26—C27—H27	117.00	H3A—C3—H3B	108.00
C28—C27—H27	117.00	C7—C8—H8	120.00
N3—C28—H28B	108.00	C9—C8—H8	120.00
C27—C28—H28A	108.00	C8—C9—H9	120.00
N3—C28—H28A	108.00	C10—C9—H9	120.00
C27—C28—H28B	108.00	C9—C10—H10	120.00
H28A—C28—H28B	108.00	C11—C10—H10	120.00
C32—C33—H33	120.00	C10—C11—H11	120.00
C34—C33—H33	119.00	C12—C11—H11	120.00
C33—C34—H34	120.00	C7—C12—H12	120.00
C35—C34—H34	120.00	C11—C12—H12	120.00
C36—C35—H35	120.00	C13—C14—H14	120.00
C34—C35—H35	120.00	C15—C14—H14	120.00
C37—C36—H36	120.00	C14—C15—H15	119.00
C35—C36—H36	120.00	C16—C15—H15	119.00
C32—C37—H37	120.00	C15—C16—H16	121.00
C36—C37—H37	119.00	C17—C16—H16	121.00
C40—C39—H39	120.00	C16—C17—H17	120.00
C38—C39—H39	120.00	C18—C17—H17	120.00
C38'—C39'—H39'	118.00	C13—C18—H18	119.00
C40'—C39'—H39'	118.00	C17—C18—H18	119.00
C41—C40—H40	120.00	C19—C20—H20	119.00
C39—C40—H40	120.00	C21—C20—H20	119.00
C41'—C40'—H40'	119.00	C20—C21—H21	120.00
C39'—C40'—H40'	119.00	C22—C21—H21	120.00
C40—C41—H41	120.00	C22—C23—H23	120.00
C42—C41—H41	120.00	C24—C23—H23	120.00
C42'—C41'—H41'	121.00	C19—C24—H24	119.00
C40'—C41'—H41'	120.00	C23—C24—H24	119.00
C43—C42—H42	120.00	O1—C25—H25A	109.00
C41—C42—H42	120.00	O1—C25—H25B	109.00
C41'—C42'—H42'	121.00	O1—C25—H25C	109.00
C43'—C42'—H42'	121.00	H25A—C25—H25B	110.00
C42—C43—H43	120.00	H25A—C25—H25C	109.00
C38—C43—H43	120.00	H25B—C25—H25C	110.00
C38'—C43'—H43'	118.00		
			
C50—O2—C47—C46	−9.7 (3)	C39—C38—C43—C42	0 (2)
C50—O2—C47—C48	170.20 (19)	C30—C38'—C39'—C40'	−179.5 (13)
C25—O1—C22—C23	7.5 (4)	C43'—C38'—C39'—C40'	−9 (2)
C25—O1—C22—C21	−172.9 (3)	C30—C38'—C43'—C42'	175.5 (12)
C31—N3—C28—C27	−85.4 (2)	C39'—C38'—C43'—C42'	5 (2)
C29—N3—C28—C27	88.6 (2)	C38—C39—C40—C41	0 (2)
C31—N3—C29—C30	−1.67 (18)	C38'—C39'—C40'—C41'	4 (2)
C31—N3—C29—C32	175.86 (15)	C39—C40—C41—C42	0 (2)
C28—N3—C31—N4	176.12 (16)	C39'—C40'—C41'—C42'	7 (2)
C28—N3—C31—C44	0.2 (3)	C40—C41—C42—C43	0 (2)
C29—N3—C31—N4	1.2 (2)	C40'—C41'—C42'—C43'	−11 (2)
C28—N3—C29—C30	−176.65 (16)	C41—C42—C43—C38	0 (2)
C28—N3—C29—C32	0.9 (3)	C41'—C42'—C43'—C38'	5 (2)
C29—N3—C31—C44	−174.72 (16)	C49—C44—C45—C46	1.2 (3)
C31—N4—C30—C38'	179.2 (8)	C31—C44—C49—C48	177.81 (18)
C30—N4—C31—N3	−0.2 (2)	C31—C44—C45—C46	−177.4 (2)
C30—N4—C31—C44	175.64 (16)	C45—C44—C49—C48	−0.7 (3)
C31—N4—C30—C38	−179.5 (8)	C44—C45—C46—C47	0.0 (3)
C31—N4—C30—C29	−0.90 (19)	C45—C46—C47—O2	178.17 (19)
C6—N1—C4—C5	0.80 (18)	C45—C46—C47—C48	−1.7 (3)
C3—N1—C6—C19	3.6 (3)	C46—C47—C48—C49	2.2 (3)
C6—N1—C4—C7	177.86 (15)	O2—C47—C48—C49	−177.69 (18)
C3—N1—C6—N2	179.59 (16)	C47—C48—C49—C44	−1.0 (3)
C4—N1—C3—C2	−91.9 (2)	C1—C2—C3—N1	−0.5 (3)
C6—N1—C3—C2	88.0 (2)	N1—C4—C5—N2	−0.81 (19)
C3—N1—C4—C5	−179.30 (15)	N1—C4—C5—C13	175.52 (17)
C3—N1—C4—C7	−2.2 (2)	C7—C4—C5—N2	−177.45 (17)
C4—N1—C6—N2	−0.53 (19)	C7—C4—C5—C13	−1.1 (3)
C4—N1—C6—C19	−176.52 (16)	N1—C4—C7—C8	94.8 (2)
C6—N2—C5—C4	0.50 (19)	N1—C4—C7—C12	−87.8 (2)
C5—N2—C6—C19	176.24 (15)	C5—C4—C7—C8	−89.1 (3)
C6—N2—C5—C13	−176.24 (16)	C5—C4—C7—C12	88.4 (3)
C5—N2—C6—N1	0.02 (18)	N2—C5—C13—C14	−171.3 (2)
C26—C27—C28—N3	−1.3 (4)	N2—C5—C13—C18	10.8 (3)
C30—C29—C32—C37	−102.5 (2)	C4—C5—C13—C14	12.7 (3)
N3—C29—C30—N4	1.60 (19)	C4—C5—C13—C18	−165.25 (19)
N3—C29—C30—C38	−180.0 (9)	N1—C6—C19—C20	155.90 (17)
N3—C29—C30—C38'	−178.5 (9)	N1—C6—C19—C24	−24.2 (3)
C32—C29—C30—N4	−175.53 (17)	N2—C6—C19—C20	−19.7 (2)
C32—C29—C30—C38	2.9 (9)	N2—C6—C19—C24	160.20 (18)
C32—C29—C30—C38'	4.4 (9)	C4—C7—C8—C9	176.6 (2)
N3—C29—C32—C33	−99.7 (2)	C12—C7—C8—C9	−1.0 (3)
N3—C29—C32—C37	80.8 (2)	C4—C7—C12—C11	−176.56 (19)
C30—C29—C32—C33	77.1 (3)	C8—C7—C12—C11	1.0 (3)
N4—C30—C38—C43	2.6 (17)	C7—C8—C9—C10	−0.2 (4)
C29—C30—C38—C39	4.8 (19)	C8—C9—C10—C11	1.3 (4)
C29—C30—C38—C43	−175.6 (9)	C9—C10—C11—C12	−1.3 (4)
N4—C30—C38'—C39'	−164.1 (12)	C10—C11—C12—C7	0.1 (4)
N4—C30—C38'—C43'	26.4 (17)	C5—C13—C14—C15	−176.0 (2)
C29—C30—C38'—C39'	16 (2)	C18—C13—C14—C15	2.0 (4)
C29—C30—C38'—C43'	−153.5 (10)	C5—C13—C18—C17	177.4 (2)
N4—C30—C38—C39	−176.9 (11)	C14—C13—C18—C17	−0.7 (3)
N3—C31—C44—C49	−68.3 (3)	C13—C14—C15—C16	−1.6 (4)
N4—C31—C44—C45	−65.1 (3)	C14—C15—C16—C17	−0.2 (4)
N4—C31—C44—C49	116.4 (2)	C15—C16—C17—C18	1.5 (4)
N3—C31—C44—C45	110.2 (2)	C16—C17—C18—C13	−1.0 (4)
C37—C32—C33—C34	0.5 (3)	C6—C19—C20—C21	−178.27 (17)
C29—C32—C37—C36	179.46 (18)	C24—C19—C20—C21	1.8 (3)
C29—C32—C33—C34	−179.1 (2)	C6—C19—C24—C23	177.41 (18)
C33—C32—C37—C36	−0.1 (3)	C20—C19—C24—C23	−2.7 (3)
C32—C33—C34—C35	−0.4 (4)	C19—C20—C21—C22	0.8 (3)
C33—C34—C35—C36	−0.1 (3)	C20—C21—C22—O1	177.75 (18)
C34—C35—C36—C37	0.5 (3)	C20—C21—C22—C23	−2.7 (3)
C35—C36—C37—C32	−0.4 (3)	O1—C22—C23—C24	−178.66 (19)
C30—C38—C39—C40	179.5 (12)	C21—C22—C23—C24	1.8 (3)
C43—C38—C39—C40	0 (2)	C22—C23—C24—C19	0.9 (3)
C30—C38—C43—C42	−179.6 (12)		

### Hydrogen-bond geometry (Å, º)

Cg1, Cg2, Cg3, Cg4 and Cg5 are the centroids of the N3/N4/C29–C31, C38'–C43', C7–C12, C13–C18 and C19-C24 rings, respectively.

**Table:**

*D*—H···*A*	*D*—H	H···*A*	*D*···*A*	*D*—H···*A*
C8—H8···*Cg*5^i^	0.93	2.87	3.585 (2)	135
C28—H28*A*···*Cg*2^ii^	0.97	2.99	3.655 (6)	127
C33—H33···*Cg*1^iii^	0.93	2.86	3.592 (2)	137
C37—H37···*Cg*1^ii^	0.93	2.96	3.789 (2)	150
C41—H41···*Cg*3^iv^	0.93	2.90	3.772 (10)	157
C41′—H41′···*Cg*3^iv^	0.93	2.85	3.705 (12)	154
C46—H46···*Cg*4	0.93	3.00	3.712 (2)	135

Symmetry codes: (i) −*x*+1/2, *y*+1/2, −*z*+1/2; (ii) −*x*+3/2, *y*−1/2, −*z*+1/2; (iii) −*x*+3/2, *y*+1/2, −*z*+1/2; (iv) *x*+1, *y*, *z*.
